# Defective proteostasis in celiac disease as a new therapeutic target

**DOI:** 10.1038/s41419-019-1392-9

**Published:** 2019-02-08

**Authors:** Luigi Maiuri, Valeria R Villella, Mauro Piacentini, Valeria Raia, Guido Kroemer

**Affiliations:** 1 Department of Health Sciences, University of Eastern Piedmont, Novara, Italy; 2 European Institute for Research in Cystic Fibrosis, San Raffaele Scientific Institute, Milan, Italy; 3 Department of Biology, University of Rome “Tor Vergata”, Rome, Italy; 4 National Institute for Infectious Diseases IRCCS ‘L. Spallanzani’, Rome, Italy; 5 Department of Translational Medical Sciences, Pediatric Unit, Regional Cystic Fibrosis Center, Federico II University Naples, Naples, Italy; 6 Equipe11 labellisée Ligue Nationale contrele Cancer, Centre de Recherche des Cordeliers, Paris, France; 7 INSERM U1138, Centre de Recherche des Cordeliers, Paris, France; 8 Université Paris Descartes, Paris, France; 9 Metabolomics and Cell Biology Platforms, Institut Gustave Roussy, Villejuif, France; 10 Pôle de Biologie, Hôpital Européen Georges Pompidou, AP-HP, Paris, France; 11 Department of Women’s and Children’s Health, Karolinska Institute, Karolinska University Hospital, Stockholm, 17176 Sweden

## Abstract

Cystic fibrosis (CF) is a disease caused by loss-of-function mutations affecting the CF transmembrane conductance regulator (CFTR), a chloride channel. Recent evidence indicates that CFTR is inhibited by a gluten/gliadin-derived peptide (P31-43), causing an acquired state of CFTR inhibition within the gut that contributes to the pathogenesis of celiac disease (CD). Of note, CFTR inhibition does not only cause intra- and extracellular ion imbalances but also affects proteostasis by activating transglutaminase-2 (TGM2) and by disabling autophagy. These three phenomena (CFTR inhibition, TGM2 activation, and autophagy impairment) engage in multiple self-amplifying circuitries, thus forming an “infernal trio”. The trio hinders enterocytes from returning to homeostasis and instead locks them in an irreversible pro-inflammatory state that ultimately facilitates T lymphocyte-mediated immune responses against another gluten/gliadin-derived peptide (P57–68), which,upon deamidation by activated TGM2, becomes fully antigenic. Hence, the pathogenic protein gliadin exemplifies a food constituent the exceptional immunogenicity of which arises from a combination of antigenicity (conferred by deaminated P57–68) and adjuvanticity (conferred by P31-43). CF can be treated by agents targeting the “infernal trio” including CFTR potentiators, TGM2 inhibitors, and autophagy enhancers. We speculate that such agents may also be used for CD therapy and indeed could constitute close-to-etiological treatments of this enteropathy.

## Facts

- Prior epithelial stress and innate immunity activation are essential for breaking oral tolerance to gliadin and triggering an (HLA) DQ2/DQ8-restricted Th1 and antibody response in celiac individuals
- How gliadin can subvert host mucosal response remains elusive
- The stress response triggered by gliadin is similar to that generated by CFTR inhibition in Cystic Fibrosis epithelia

## Open questions

- Does CFTR inhibition mediate stress response in gliadin sensitive epithelial cells?
- Does CFTR inhibition derail proteostasis upon gliadin exposure?
- How do CFTR, TGM2, and autophagy contribute to sustain gliadin-induced immunopathology?

## A brief overview of celiac disease (CD)

Celiac disease (CD) affects up to 1% of the world population. This enteropathy is triggered by an immunogenic/autoimmune reaction against gluten, and in particular its component gliadin, that is contained in wheat, barley, rye, and related species of cereals^1-3^. After its ingestion, gliadin is subjected to partial proteolysis to generate peptides that, instead of being ignored by the immune system or triggering oral tolerance, induce an immunogenic and autoimmune reaction that causes intestinal inflammation and eventually culminates in villous atrophy with consequent malabsorption^4–12^. Although the disease often responds to the dietary avoidance of gluten-containing food items, it can evolve to refractory CD, meaning that the pathology self-perpetuates in spite of a one year-long strictly gluten-free regimen^13,14^.

### Gliadin molecular features underlying CD pathogenesis

**Fig. 1:**
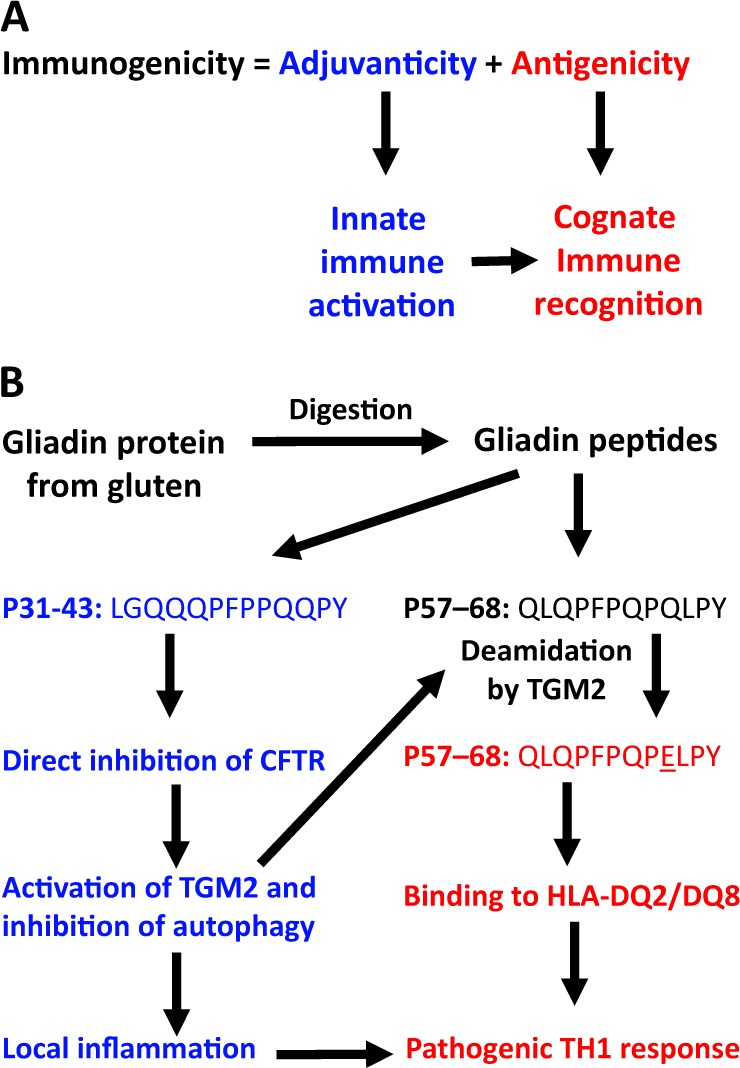
Principles of the immunogenic action of gliadin. **a** General rules governing immunogenicity. **b** Gliadin-derived peptides that act as an adjuvant (P31-P43) or as an immunogen (P57–68). Note that innate immune reactions are required for full cognate immune responses. This is exemplified by the activation of transglutaminase-2 (TGM2), downstream of the inhibition of cystic fibrosis transmembrane receptor (CFTR), as well as local inflammation that perturbs tissue homeostasis, increases intestinal permeability, and attracts immune cells, thus enabling to launch a TH1 immune response against deamidated P57–68

As for any kind of immune response, the immunogenicity of gliadin results from a combination of antigenicity and adjuvanticity^15,16^ (Fig. 1a). These two properties correspond to distinct moieties of the protein. Antigenicity is conferred by a 33 amino acids-long peptide (P55–87) and its fragment QLQPFPQPQLPY (P57–68) that is deamidated by transglutaminase-2 (TGM2), upon its activation, to yield QLQPFPQPELPY (in which one glutamine [Q] residue, Q65, has been converted into glutamic acid [E]) and then binds to one particular MHC class II type (in particular HLA-DQ2/DQ8), meaning that only individuals bearing such HLA alleles are genetically susceptible to CD^4,6^. In a subset of these individuals, P57–68 induces a pathogenic T helper 1 (TH1) response that leads to the immune-mediated destruction of intestinal epithelial cells^5,9,10^. Adjuvanticity is conferred by a 25-mer (P31–55) and its fragment LGQQQPFPPQQPY (P31–43) that is not recognized by T lymphocytes and instead damages the small intestine to create local inflammation and to initiate a series of vicious cycles that increase gut permeability so that gliadin and its fragments can perturb the ecosystem composed by enterocytes and a variety of immune cells in the intestinal wall^5,9,10^. Schematically (Fig. 1b), it appears that the gliadin-derived adjuvant peptide P31–43 provides the initial signal of the cascade by perturbing the physiology of enterocytes, leading to the secretion of pro-inflammatory cytokines such as interleukin-1β (IL1β) and interleukin-15 (IL-15) as signs of an innate immune response. These adjuvant signals then condition the local microenvironment to facilitate the subsequent cognate immune response against the immunogenic peptide P57–68^5,9,10^.

## New molecular mechanisms underlying the CD-cystic fibrosis (CF) connection

### Gliadin-derived P31-43 and CFTR

**Fig. 2:**
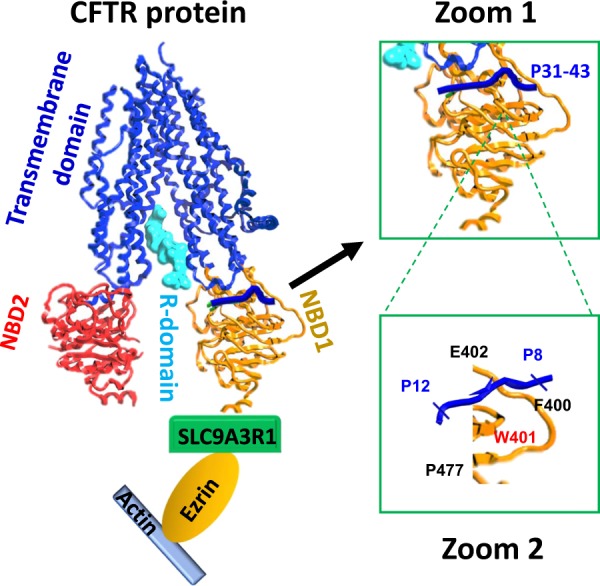
Molecular model of the interaction between gliadin peptide P31-43 and CFTR. On the left, the entire CFTR protein with its domain organization, as well as its interaction with the actin cytoskeleton (mediated by solute carrier family 9 (sodium/hydrogen exchanger), isoform 3 [SLC9A3R1] and ezrin) is shown. Zoom 1 demonstrates the interaction of P31-43 from gliadin with the nucleotide binding domain-1 (NBD1), while Zoom 2 details the amino acids within P31-43 and CFTR that are involved in the binding of P31-43 to NBD1

Recently, we reported the discovery that P31–43 acts on a specific receptor expressed on the surface of enterocytes, namely, cystic fibrosis transmembrane conductance regulator (CFTR), the chloride channel that is mutated in cystic fibrosis (CF)^17^. Indeed, we observed that children with CF (in which CFTR loses its expression or function) have a three-fold increased risk to develop CD^17–20^. Mice bearing a knockout of CFTR (genotype: *Cftr*^*−/−*^) or a loss-of-function mutation frequently occurring in human CF (genotype: *Cftr*^F508del/F508del^) exhibited signs of intestinal inflammation, including a local increase in *IL-15* mRNA expression, that could be increased by oral challenge with gliadin (in *Cftr*^F508del/F508del^ mice). More convincingly, we could demonstrate a direct molecular and functional interaction between P31–43 and CFTR using a variety of methods, namely, (i) computer-assisted calculations, (ii) plasmon surface resonance, (iii) co-immunoprecipitation, (iv) co-localization of immunofluorescence signals, (v) and functional inhibition of CFTR-dependent chloride fluxes in P31–43–treated cells or the intestine of gliadin-exposed mice^17^. These assays, which were refined with the help of mutated P31-43 peptides, allowed us to define the molecular interaction between P31-43 and CFTR at the amino acid level. According to our molecular model (Fig. 2), P31-43 binds to a particular portion of CFTR, the nucleotide binding domain-1 (NBD1). This domain is responsible for the ATPase activity of CFTR (which is inhibited by P31-43) and exhibits two distinct folds, correlating with the opening and closing state of the chloride channel. Apparently, P31-43 can only bind to NBD1 when it is in the closing-associated conformation, hence blocking its gating function. Of note, when CFTR is stimulated by Ivacaftor, a pharmacological “potentiator” that favors the opening of the chloride channel, P31-43 does not interact any more with CFTR, corroborating the model^17^.

### TGM2 activation and autophagy

As mentioned before, CFTR is the protein that is mutated in CF^21,22^. Logically, defective chloride channel function compromises the capacity of epithelial cells to transport chloride. This explains the reason why the sweat from CF patients is abnormally salty, giving rise to the diagnostic “sweat test” in which chloride concentrations are measured after dermal stimulation of sweat glands with the parasympathomimetic pilocarpine^23–25^. More importantly, the mucus produced by respiratory epithelial cells becomes abnormally viscous, facilitating recurrent pulmonary infections by opportunistic bacteria with the consequent permanent loss of respiratory function^21,26,27^. Similar mechanic issues can lead to obstruction of the pancreatic ducts with consequent gastrointestinal problems^21,28,29^. Nonetheless, the loss-of-function of CFTR chloride channel function goes well beyond the problem of excessive extracellular chloride concentrations. Indeed, when CFTR is disabled, the intracellular milieu undergoes major pathogenic changes. On one side, CFTR inhibition results in the activation of TGM2 (refs. ^30–32^), an enzyme that can deaminate P57–68 to introduce a posttranslational modification increasing its antigenicity^1,2,5,9,10^. Incidentally, TGM2 is one of the major targets of autoantibodies in CD to the point that the detection of TGM2-specific autoreactivity is pathognomonic for CD^4,6,33^. In addition, TGM2 activation causes the protein Beclin 1 (BECN1) to be sequestered at the endoplasmic reticulum and to be reduced in its abundance^32,34,35^. BECN1 nucleates a multiprotein complex (the phosphatidylinositol-3-kinase (PI3K) complex-3) that is required for autophagy and some routes of endosomal trafficking, thus perturbing cellular proteostasis^36^. Autophagy is (one of) the most important proteostasis mechanisms in thus far that it constitutes the sole mechanism to degrade large protein complexes and even entire cytoplasmic organelles, assuring their removal (to reduce their toxicity) and their replacement (to mediate cellular repair and rejuvenation)^37^.

### The “infernal trio”

**Fig. 3:**
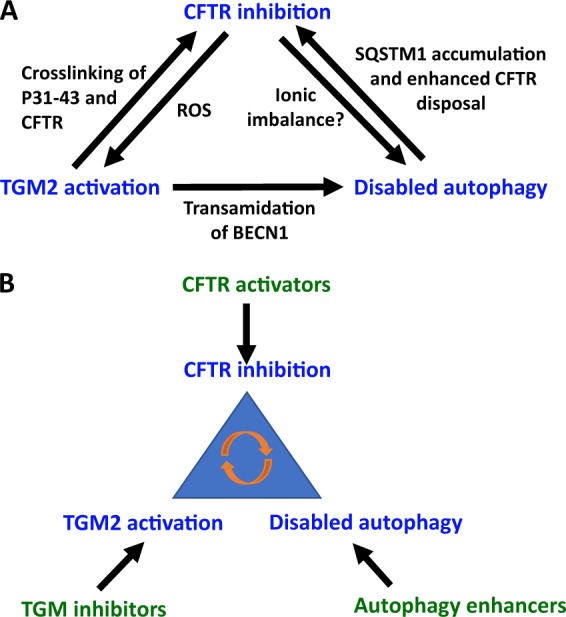
The “infernal trio”. **a** Feedforward circuits explaining the mutual amplification between CFTR inhibition, transglutaminase-2 (TGM2) activation and disabled autophagy. **b** Pharmacological approaches to act on the “infernal trio”. Note that CFTR activators, TGM inhibitors and autophagy enhancers act on a highly connected node of self-amplifying circuitries, meaning that they may mediate synergistic effects on the system

In sum, CFTR inhibition causes two major alterations in cellular function, TGM2 activation and autophagy inhibition. In a certain sense, these three features of CF and CD (in which CFTR is inhibited due to mutations or oral gliadin uptake, respectively) engage in several pathogenic feedforward loops giving rise to an “infernal trio” (inhibited CFTR, activated TGM2, disabled autophagy) (Fig. 3a). For example, TGM2 can crosslink CFTR and the adjuvant peptide P31-43 as it interacts with CFTR to create a trimolecular complex involving CFTR, P31-43 and TGM2, thus rendering CFTR inhibition irreversible^17^. In addition, disabled autophagy causes the accumulation of a particular protein, sequestosome-1 (SQSTM1) that can interact with ubiquitinylated CFTR, favoring its lysosomal degradation^32,34,35,38,39^. This means that cells in which CFTR is inhibited progressively derail into a spiral in which CFTR is permanently inactivated, irreversibly locking them in a state of perturbed proteostasis, mostly due to the constant activation of TGM2 and the inactivation of autophagy. Especially this latter impairment favors a pro-inflammatory state due to deficient clearance of potentially harmful protein complexes and aging organelles^40^, ultimately resulting in the stimulation of the NFκB pathway and activation of the NLRP3 inflammasome^41–43^. This causes gliadin-exposed, CFTR-inhibited enterocytes to produce pro-inflammatory cytokines, in particular IL-15 (downstream of NFκB) and IL1β (downstream of both NFκB and the NLRP3 inflammasome)^11,17^. In addition, TGM2 activation and autophagy inhibition cause a reorganization of the actin cytoskeleton that may explain, or contribute to, the enfeeblement of the gut barrier function relying on tight junctions between enterocytes^10,44,45^. Increased gut permeability, in turn, may facilitate the access of gliadin-derived peptides to the intestinal wall (including that of antigenic P57–68) and favor the translocation of intestinal bacteria or bacterial products (such as lipopolysaccharide, LPS), thereby promoting the activation of pattern recognition receptors (such as Toll-like receptors, in particular TLR4 for LPS) and accentuating the inflammatory state^46^.

## New possible therapeutic strategies against CD

### Current strategies under development

How can this pathogenic cascade then be interrupted? Beyond the obvious solution to prevent antigen exposure (by means of a gluten-free diet or the still experimental provision of enzymes that degrade pathogenic gliadin-derived peptides in the gut lumen)^47,48^ a number of distinct strategies can be envisaged. Several biotech companies are working on the concept of tolerogenic vaccines to “desensitize“ patients vis-à-vis of gliadin, thus switching the immunogenic/autoimmune reaction into a response that reinforces immunosuppressive circuitries to assure permanent tolerance of the xenogenic antigen^49,50^. Others are developing agents designed to prevent the leaky gut syndrome that participates to CD pathogenesis, for instance by providing synthetic peptides that improve the maintenance of tight junction by enterocytes^51–53^.

### Targeting the infernal trio

Based on our recent work, we suggest three additional strategies that might intercept central mechanisms of CD and that target the aforementioned “infernal trio” (Fig. 3b). First, it may be possible to use agents such as Ivacaftor to increase the chloride channel function of unmutated CFTR in CD and to reduce the interaction of CFTR with the gliadin-derived adjuvant P31-43 (ref. ^17^). Second, it can be envisaged to inhibit TGM2, either with cysteamine or other, more specific agents that are currently in development^17,30–32,34,38,44,54^. Third, it can be attempted to stimulate autophagy. For this latter purpose, a number of options are available. Given the general rule in pharmacology that antagonists (inhibitors) have higher prospect of mediating positive effects at the expense of controllable side effects than agonists (activators)^55^, we believe that the best option to induce autophagy is to interfere with the function of its endogenous blockers including the acetyltransferase EP300 (examples: aspirin, epigallocatechin gallate, EGCG, and spermidine)^56–58^, the BECN1 inhibitory proteins from the BCL2 family (examples: ABT737, navitoclax, venetoclax)^59^, and the mechanistic target of rapamycin complex-1 (mTORC1; examples: rapamycin, everolimus, tacrolimus)^55^.

### Possible drug repurposing in CD

It is important to note that each of the aforementioned possible strategies for the treatment of CD (whose primary cause apparently is the gluten-mediated inhibition of CFTR) has been successfully applied to CF (which is due to inherited loss-of-function mutations of CFTR). First and foremost, Ivacaftor has initially been designed for improving the function of specific CFTR mutants in CF and is right now FDA-approved for the treatment of CF caused by a panel of CFTR mutations that altogether represent 5-10 percent of all CF cases^60–64^. Second, cysteamine (an FDA-approved agent used for the treatment of cystinosis) can be used to inhibit TGM2 and actually prevents the intestinal obstruction that frequently causes the death of mice bearing the *Cftr*^F508del/F508del^ mutation, while it partially rescues the expression and function of the mutant CFTR protein on enterocytes in vivo, phenocopying the effect of the knockout of *Tgm2* (refs. ^25,54,65^). Similarly, cysteamine can restore the expression of mutant CFTR protein at the plasma membrane of cultured cells from patients with the *Cftr*^F508del/F508del^ mutation^25,38,65–68^. Third, the autophagy activator EGCG can be combined with cysteamine to improve and prolong its rescuing effect^25,64^. In *Cftr*^F508del/F508del^ mice, restoration of CFTR function by a combination with cysteamine plus EGCG is lost when autophagy is inhibited due to haploinsufficiency of BECN1 (genotype: *Becn1*^+/−^), confirming that autophagy is required for optimal therapeutic efficacy^25,66^. Moreover, two independent phase-2 clinical trials demonstrated that CF patients bearing the CFTR-F508 mutation responded to a combination of cysteamine plus EGCG by an improvement of CFTR function (as determined by the sweat test and by measuring plasma membrane expression and function of the mutated CFTR protein in respiratory epithelia), as well as by a reduction of lung inflammation (as indicated by a decline in inflammatory cytokines in the sputum)^25,65^. These findings illustrate the clinical feasibility of tackling the “infernal trio”.

## Conclusions

Given the nosological similarities of CF and CD, which are due to inherited and acquired CFTR inhibition, respectively, it is tempting to apply the lessons learned in one disease to the other. Thus, it will be crucial to examine the possibility to combine Ivacaftor (or other CFTR potentiators) with cysteamine (or other yet-to-be-developed TGM2 inhibitors) and EGCG (or alternative autophagy enhancers) with the scope of interrupting the self-enforcing circuitry that likely intervenes in CD. It remains to be clinically demonstrated that such interventions work. Moreover, it will be important to explore the possibility of combining such close-to-etiological treatments with suitable life style interventions to avoid excessive gluten/gliadin uptake, as well as non-specific measures designed to dampen inflammation and to improve the gut barrier function. Enticed by these perspectives, we anticipate that novel approaches to treat CD will soon reach the clinics.

Box 1 Molecular basis for the detrimental effects of gliadin peptides in intestinal epithelial cells

- The gliadin-derived peptide P31-43 binds to specific residues of the NBD1 domain, provided that CFTR is in the inactive conformation. P31-43 and ATP compete for the same binding site of NBD1, meaning that P31-43 impairs CFTR function in intestinal epithelial cells.
- Inhibition of CFTR function disrupts proteostasis and induces oxidative stress with consequent persistent TGM2 activation. As a result, TGM2 is recruited to a trimolecular complex that also involves CFTR and P31-43, stabilizing the interaction between these two molecules and worsening CFTR inhibition.
- TGM2 activation inhibits the BECN1 complex, leading to derangement of endosomal trafficking, accumulation of SQSTM1 and cytoskeleton disassembly.
- P31-43-mediated CFTR inhibition leads to inflammasome activation, resulting in IL1β secretion, as well as in NF-κB activation and consequent IL-15 production.
- Stressed enterocytes stimulate local inflammation that generates favorable conditions for HLA-DQ2/DQ8-restricted immune responses against immunodominant gliadin peptides, thus triggering celiac disease.
- By increasing the probability of CFTR channel opening, CFTR potentiators can avoid P31-43 binding to CFTR and ultimately prevent the gliadin-induced immunopathology.
